# Particulate matter and risk of parkinson disease in a large prospective study of women

**DOI:** 10.1186/1476-069X-13-80

**Published:** 2014-10-07

**Authors:** Natalia Palacios, Kathryn C Fitzgerald, Jaime E Hart, Marc G Weisskopf, Michael A Schwarzschild, Alberto Ascherio, Francine Laden

**Affiliations:** Department of Nutrition, Harvard School of Public Health, Boston, Massachusetts USA; Channing Division of Network Medicine, Department of Medicine, Brigham and Women’s Hospital and Harvard Medical School, Boston, Massachusetts USA; Department of Neurology, Massachusetts General Hospital, Boston, Massachusetts USA; Department of Environmental Health, Harvard School of Public Health, Boston, Massachusetts USA; Department of Epidemiology, Harvard School of Public Health, Boston, Massachusetts USA

**Keywords:** Epidemiology, Cohort studies, Incidence studies, Parkinson disease/Parkinsonism

## Abstract

**Background:**

Exposure to air pollution has been implicated in a number of adverse health outcomes and the effect of particulate matter (PM) on the brain is beginning to be recognized. Yet, no prospective study has examined the association between PM and risk of Parkinson Disease. Thus, our goal was assess if exposure to particulate matter air pollution is related to risk of Parkinson’s disease (PD) in the Nurses’ Health Study (NHS), a large prospective cohort of women.

**Methods:**

Cumulative average exposure to different size fractions of PM up to 2 years before the onset of PD, was estimated using a spatio-temporal model by linking each individual’s places of residence throughout the study with location-specific air pollution levels. We prospectively followed 115,767 women in the NHS, identified 508 incident PD cases and used multivariable Cox proportional hazards models to estimate the risk of PD associated with each size fraction of PM independently.

**Results:**

In models adjusted for age in months, smoking, region, population density, caffeine and ibuprofen intake, we observed no statistically significant associations between exposure to air pollution and PD risk. The relative risk (RR) comparing the top quartile to the bottom quartile of PM exposure was 0.99 (95% Confidence Intervals (CI): 0.84,1.16) for PM_10_ (≤10 microns in diameter), 1.08 (95% CI: 0.81, 1.45) for PM_2.5_ (≤2.5 microns in diameter), and 0.92 (95% CI: 0.71, 1.19) for PM_10–2.5_ (2.5 to 10 microns in diameter).

**Conclusions:**

In this study, we found no evidence that exposure to air pollution is a risk factor for PD.

## Background

Chronic exposure to air pollution has detrimental effects on human health [1–3]. However, little is known about the effects of air pollution on neurological outcomes and risk of Parkinson’s disease (PD). The existence of a link between air pollution and PD risk is suggested by the observations that toxins in air pollution evoke a systemic inflammatory response and oxidative stress [4], markers of which have been shown in some studies to be elevated among PD patients [5]. The brain is particularly vulnerable to oxidative stress, due it’s high consumption of oxygen, low level of antioxidants, high levels of polyunsaturated fatty acids and elevated iron content (particularly in the substantia nigra, a key brain area for PD) [6]. Further, increased plasma levels of inflammatory markers have been associated in some studies [7, 8], whereas higher urate, a potent antioxidant, appears to have neuroprotective effects in experimental models [9, 10] and is associated with lower PD risk [11–13]. In a recent analysis, Willis et al. linked Environmental Protection Agency (EPA) data on lead, copper and mercury emissions with the Medicare dataset on the county level throughout the US, and showed increased risk of PD among participants living in urban counties with the highest reported emissions of copper and manganese [14]. An adverse effect of air pollution would be consistent with evidence that in some cases PD may originate in the olfactory bulb and may thus be caused by a pathogen that enters the body through the nasal pathway [15, 16]. We thus examined in a large prospective study of US nurses, whether exposure to ambient PM_10_, PM_2.5_ and PM_10–2.5_ is associated with increased risk of PD.

## Methods

We examined the effects on PD risk of exposure to particulate matter less than or equal to 10 microns (PM_10_), less than or equal to 2.5 microns (PM_2.5_), and between 2.5 and 10 microns in diameter (PM_10–2.5_) in a large prospective cohort of older women with biennially updated addresses living throughout the contiguous United States. This study was approved by the Brigham and Women’s Hospital IRB.

### Study population

Since 1976, 121,700 female registered nurses have been participating in the Nurses’ Health Study (NHS). The participants were recruited from 11 states (California, Connecticut, Florida, Maryland, Massachusetts, Michigan, New York, New Jersey, Pennsylvania, Ohio and Texas) and were between 30 and 55 years old at baseline. At baseline and every two years thereafter, participants received, at their residential address, questionnaires asking about lifestyle factors and health outcomes. A question regarding PD was first asked in 1994. The follow-up rate in the NHS has been above 90% for each follow-up cycle. Detailed description of the study cohort is provided elsewhere [17]. The Institutional Review Board (IRB) at the Brigham and Women’s Hospital approved this study.

### Exposure assessment

Monthly exposures to ambient air pollution were estimated for each participant using spatio-temporal models, discussed in detail elsewhere [18–20]. In brief, generalized additive models of PM_10_ from 1988 through 2007 were developed [19] for the continental United States (US), using monthly average PM_10_ data from EPA’s Air Quality System (AQS), a nationwide network of continuous and filter-based monitors, as well as data from various other sources, including the Interagency Monitoring of Protected Visual Environments (IMPROVE) network, and several Harvard-based research studies. A geographic information system (GIS) was used to derive the following model covariates: population density, distance to nearest road, elevation, and urban land use. The estimation of PM_2.5_ was similar; however, because EPA AQS monitoring data for PM_2.5_ was not available prior to 1999, separate models were created for pre- and post-1999 PM_2.5_. To estimate PM_2.5_ prior to 1999, the model relied on measured PM_10_ pre-1999 and the PM_2.5_ to PM_10_ ratio from the spatio-temporal model post-1999, as well as estimated extinction coefficients from airport visibility data. PM_10–2.5_ was estimated by subtracting values for PM_2.5_ from those for PM_10_. All models showed little bias and a high degree of precision when evaluated with a cross-validation approach, where a sub-section of the monitors were held out to compare predicted and observed values [19, 20].

### PD Ascertainment

The method used to ascertain PD cases has been described previously [21]. Briefly, since 1994, new cases of PD in the NHS have been identified through biennial questionnaires. When a participant reports PD, consent is sought from the participant to contact their treating neurologist (or internist if the neurologist is not available) and upon receipt of the consent the doctor is contacted to complete a questionnaire to confirm the diagnosis of PD and send a copy of the medical records. Of the self-reported PD cases in the NHS, we were able to contact 86% and of those, 69% provided consent to review medical records (17% did not give permission and 14% denied the diagnosis of PD). The medical records are reviewed by a movement disorder specialist (M.A.S.) blinded to the exposure status. A case is considered confirmed if the treating physician reported it as either definite or probable, or if the medical record included evidence of either a final diagnosis of PD made by a neurologist or evidence of two or more of the three cardinal signs of PD (bradykinesia, rigidity, rest tremor) in the absence of characteristics suggesting an alternate diagnosis. The main analyses were performed among confirmed cases. We performed sensitivity analyses including participants who reported PD but did not give consent to review their medical records. Of the 508 incident PD cases included in this study, 212 were definite and 296 were probable cases.

### Statistical analysis

Cox proportional hazards models with age as the time scale were used to model the association between exposure to PM_10,_ PM_2.5,_ PM_10–2.5,_ and PD. Person-months of follow-up were calculated from baseline (June 30, 1990, to allow for a 2-year lag prior to the first incident PD cases reported in this study) through the end of follow-up (June 30, 2008), death or date of PD diagnosis, whichever occurred earlier. Exposure to air pollution was included in the models as a time-dependent variable that was incremented each month. We focused on the cumulative average exposure to PM_10_, PM_2.5_ and PM_10–2.5_ up to 2 years before PD diagnosis to best capture any effects of air pollution before the onset of PD. In separate models, we also considered a lag of 5 years prior to diagnosis, as well as using PM exposure in 2000, when the PM data is most complete (results not shown). Analyses were adjusted for age in months, region of the United States (northeast,/midwest/west/south), pack years smoking, smoking status (never/past/current), population density, caffeine consumption and use of ibuprofen. Regarding ibuprofen use, our questionnaire asked the following question: “On average, how many days each month do you take the following medicine (ibuprofen)”, and the responses were never, 1–4 days, 5–14 days, 15–21 days, and 22 or more days. Participants taking the drug 1 or more days per month were considered ever users for the purpose of this study. We also conducted additional analyses adjusting for tract-level income and housing value, as taken from the 2000 US census [22]. These were included in models as time-dependent variables. Because dietary, lifestyle and geographic factors may modify the relationship between air pollution and PD, we conducted additional analyses, stratified by smoking, caffeine intake and ibuprofen use, the main known factors related to PD risk, as well as region of the US (as the pollutant type and thus the effect on PD may differ by region). For tests of trend, the median value in each quintile was used as a continuous variable to allow for nonlinear associations. SAS version 9.1 (SAS Institute, Cary, NC, USA) was used for the statistical analyses.

## Results

Table 1 shows the baseline characteristics of the study population. There were 115,620 participants in the study at baseline, and 508 incident PD cases included in prospective analyses. The characteristics of the PD cases included in the study were as follows: the mean age at PD onset was 71 years, 30% were current smokers at baseline, 28% were ever users of ibuprofen (using one or more times per month), and the mean baseline caffeine among intake was 231 mg/day. The average follow-up for participants included in the analyses was 16.6 years (SD: 3.5 years). All three measures of air pollution were positively associated with population density. PM_10_ was highly correlated with both PM_2.5_ and PM_10–2.5_ (r = 0.73 and 0.82); however, PM_2.5_ and PM_10–2.5_ were not highly correlated (r = 0.26).

**Table 1:** **Age**-**standardized characteristics of the 111**,**769 study participants at baseline in 1990 with respect to categories of PM**_**10**_

	PM _10_			
Pollution category	Q1*	Q2	Q3	Q4
Median PM^a^	19.0	22.6	26.0	31.8
Age, mean (sd)	58.2 (7.2)	58.2 (7.2)	58.1 (7.2)	58.2 (7.2)
Never Smoker (%)	48.0	46.5	45.9	42.3
Pack years, mean (sd)	12.8 (19.3)	13.1 (19.4)	13.0 (19.1)	13.9 (19.5)
Caffeine mg/day, mean (sd)	284.2			
(239.5)	284.6			
(238.1)	280.2			
(234.1)	274.8			
(228.2)				
Ibuprofen ever user (%)	28.7	28.4	28.7	26.8
Census tract income^b^, USD, mean (sd)	51602.9			
(16926)	58044.3			
(22510.2)	66772.3			
(26322.7)	66,772.3			
(26322.7)				
(Population density)^b^, (persons/tract), mean, (sd)	1436.3			
(2184.5)	2139.3			
(3216.8)	1922.6			
(3088.5)	4449.4			
(9575.1)				
Region of US				
‘Northeast Region	12.2	18.5	41.8	61.2
‘Midwest region’, %	31.8	26.2	27.2	14.7
‘West region’, %	7.0	3.5	6.7	15.9
‘South region’, %	49.0	51.8	24.3	8.2

*Q (1,2,3,4) - quartiles – categories (C1, C2, C3 and C4) for distance to road as shown in Table 3.

g/day: grams/day.

^a^μg/m^3^ (PM).

^b^tract-level variable based on the 2000 US Census.

We found no statistically significant associations between air pollution and PD risk. The relative risk (RR) comparing the top quartile to the bottom quartile of PM exposure was 0.99 (95% Confidence Intervals (CI): 0.84,1.16) for PM_10_, 1.08 (95% CI: 0.81, 1.45) for PM_2.5,_ and 0.92 (95% CI: 0.71, 1.19) for PM_10–2.5_. These results are shown in Table 2.

**Table 2:** **Exposure to PM**_**10** ,_ **PM**_**2.5** ,_ **and PM**_**10**–**2.5**_ **and risk of PD in the Nurses**’ **Health Study**, **1990**–**2008** (**N** = **111**,**769 women at baseline**)

			Age adjusted		Multivariate ^a^		
	PY	Cases	RR	95% CI	RR	95% CI	P-trend
**Quartiles of PM**_**10**_							
3.8-21.0 μg/m^3^	484444	141	1.00	Ref	1.00	Ref	
21.0– 24.3 μg/m^3^	484483	135	1.06	(0.83, 1.34)	1.09	(0.86, 1.39)	
24.3 – 28.3 μg/m^3^	484520	120	1.02	(0.80, 1.30)	1.03	(0.80, 1.32)	
28.3 – 88.3 μg/m^3^	484550	112	1.03	(0.80, 1.32)	1.03	(0.78, 1.37)	0.92
**Continuous^** **PM**_**10**_	1937996	508	0.99	(0.86, 1.12)	0.99	(0.84, 1.16)	
**Quartiles of PM**_**2.5**_							
1.2-12.6 μg/m^3^	484429	131	1.00	Ref	1.00	Ref	
12.6-15.0 μg/m^3^	484482	131	1.12	(0.88, 1.43)	1.07	(0.83, 1.38)	
15.0-17.4 μg/m^3^	484513	137	1.23	(0.97, 1.57)	1.14	(0.88, 1.48)	
17.4-73.9 μg/m^3^	484573	109	1.17	(0.90, 1.51)	1.10	(0.83, 1.45)	0.43
**Continuous^** **PM**_**2.5**_	1937996	508	1.17	(0.90-1.51)	1.08	(0.81-1.45)	
**Quartiles of PM**_**10**–**2.5**_							
0-7.2 μg/m^3^	484479	149	1.00	(Ref)	1.00	(Ref)	
7.2-9.2 μg/m^3^	484496	121	0.87	(0.69, 1.11)	0.91	(0.71, 1.16)	
9.2-12.0 μg/m^3^	484495	119	0.87	(0.68, 1.11)	0.93	(0.72, 1.20)	
12.0-67.1 μg/m^3^	484506	119	0.87	(0.68, 1.11)	0.93	(0.69, 1.26)	0.64
**Continuous^** **PM**_**10**–**25**_	1937996	508	0.90	(0.74-1.09)	0.92	(0.71-1.19)	

*PY*: person years; *RR*: relative risk; 95% CI: 95 percent confidence interval.

^a^adjusted for age, smoking, region (northeast, midwest, west, and south), population density, caffeine intake (<100 mg/day vs. over 100 mg/day) and ibuprofen use (ever/never user).

^per 10 μg/m^3^.

In stratified analyses, we did not observe any statistically significant interactions with smoking caffeine consumption and PM exposure (Table 3). The effect of PM on PD risk did not differ significantly by region of the US.

**Table 3:** **Exposure to PM and risk of PD in the Nurses**’ **Health Study**, **1990**–**2008** (**N** = **111**,**769**) **by smoking status**, **caffeine intake and ibuprofen use**

	PY	Cases	RR ^a^	95% CI	PY	Cases	RR ^a^	95% CI	p-int
By smoking status:	Never smokers				Ever smokers				
**Quartiles of PM**_**10**_									
Q1	206697	73	1.00	Ref	278761	68	1.00	Ref	
Q2	209774	75	1.14	(0.82, 1.58)	275654	60	1.03	(0.72, 1.47)	
Q3	213834	63	0.99	(0.70, 1.41)	271618	57	1.05	(0.73, 1.52)	
Q4	213834	56	0.92	(0.62, 1.36)	265933	56	1.14	(0.76, 1.71)	0.31
Continuous* **PM**_**10**_	849835	267	0.96	(0.85, 1.09)	1091965	241	1.04	(0.92, 1.18)	0.42
**Quartiles of PM**_**2.5**_									
Q1	210817	69	1.00	Ref	274637	62	1.00	Ref	
Q2	204967	69	1.07	(0.76, 1.52)	280601	62	1.06	(0.74, 1.54)	
Q3	214387	70	1.07	(0.74, 1.53)	271003	67	1.21	(0.84, 1.76)	
Q4	219664	59	1.06	(0.72, 1.55)	265724	50	1.12	(0.74, 1.69)	0.58
Continuous* **PM**_**2.5**_	849835	267	1.01	(0.90, 1.15)	1091965	241	1.05	(0.92, 1.19)	0.66
**Quartiles of PM**_**10–2.5**_									
Q1	212002	85	1.00	Ref	273461	64	1.00	Ref	
Q2	204860	60	0.79	(0.57, 1.11)	280562	61	1.05	(0.74, 1.51)	
Q3	210385	58	0.77	(0.54, 1.09)	274925	61	1.14	(0.79, 1.65)	
Q4	222580	64	0.82	(0.54, 1.24)	263007	55	1.05	(0.67, 1.65)	0.26
**Continuous^** **PM**_**10–25**_	849835	267	0.93	(0.81, 1.05)	1091965	241	1.03	(0.90, 1.18)	0.41
**By Caffeine intake:**	**<100 mg/day caffeine**				**> = 100 mg/day caffeine**				
**Quartiles of PM**_**10**_									
Q1	196761	69	1.00	Ref	287683	72	1.00	(Ref)	
Q2	189470	69	1.20	(0.85, 1.68)	295013	66	0.99	(0.70, 1.40)	
Q3	189270	73	1.35	(0.95, 1.91)	295251	47	0.74	(0.50, 1.08)	
Q4	184296	57	1.20	(0.81, 1.78)	300254	55	0.87	(0.60, 1.31)	0.23
Continuous* PM_**10**_	759796	268	1.08	(0.95, 1.22)	1178200	240	0.93	(0.81, 1.06)	0.49
**Quartiles of PM**_**2.5**_									
Q1	194876	65		(Ref)	289553	66	1.00	(Ref)	
Q2	189312	68	1.02	(0.71, 1.46)	295169	63	0.77	(0.53, 1.10)	
Q3	194935	74	1.06	(0.73, 1.55)	289578	63	0.86	(0.60, 1.24)	
Q4	180673	61	0.88	(0.58, 1.32)	303900	48	0.69	(0.44, 1.09)	0.22
Continuous* **PM**_**25**_	759796	268	0.97	(0.85, 1.10)	120299	240	0.91	(0.79, 1.04)	0.38
**Quartiles of PM**_**10–2.5**_									
Q1	196694	76	1.00	(Ref)	287785	73	1.00	(Ref)	
Q2	185789	67	1.05	(0.75, 1.47)	298707	54	0.77	(0.53, 1.10)	
Q3	188793	59	0.99	(0.70, 1.43)	295702	60	0.86	(0.60, 1,24)	
Q4	188513	66	1.20	(0.79, 1.80)	295993	53	0.69	(0.44, 1.09)	0.45
**Continuous^** **PM**_**10–25**_	759796	268	1.04	(0.92, 1.19)	1178200	240	0.91	(0.79, 1.04)	0.72
**By use of ibuprofen**	**Never user**				**Ever user**				
**Quartiles of PM**_**10**_									
Q1	378733	101	1.00	(Ref)	105711	40	1.00	(Ref)	
Q2	375957	110	1.22	(0.93, 1.61)	108526	25	0.76	(0.45, 1.26)	
Q3	373161	87	1.04	(0.77, 1.40)	111359	33	1.03	(0.63, 1.68)	
Q4	369737	95	1.20	(0.87, 1.65)	114813	17	0.62	(0.33, 1.16)	0.04
Continuous* **PM**_**10**_	1491149	375	1.04	(0.04, 1.15)	440409	115	0.91	(0.75, 1.10)	0.03
**Quartiles of PM**_**2.5**_									
Q1	377642	101	1.00	(Ref)	106787	30	1.00	(Ref)	
Q2	376538	103	1.16	(0.87, 1.55)	107943	28	0.81	(0.47, 1.37)	
Q3	375892	98	1.13	(0.84, 1.52)	108622	39	1.14	(0.68, 1.91)	
Q4	367516	91	1.26	(0.87, 1.55)	117056	18	0.64	(0.34, 1.20)	0.43
Continuous* **PM**_**25**_	1491149	393	1.07	(0.97, 1.18)	440409	115	0.93	(0.77, 1.12)	0.38
**Quartiles of PM**_**10–2.5**_									
Q1	375666	107	1.00	Ref	108813	42	1.00	(Ref)	
Q2	374592	90	0.92	(0.70, 1.23)	109905	31	0.81	(0.50, 1.30)	
Q3	375149	98	1.02	(0.77, 1.35)	109346	21	0.56	(0.33, 0.96)	
Q4	372167	98	1.01	(0.76, 1.36)	112339	21	0.55	(0.32, 0.97)	0.01
**Continuous^** **PM**_**10**–**25**_	1497588	393	1.01	(0.92, 1.11)	440409	115	0.80	(0.67, 0.96)	0.01

*Abbreviations*: *PY* person years; *RR* relative risk; *95% CI* 95 percent confidence interval.

^a^adjusted for age, smoking (except in smoking stratified analyses), region (northeast, midwest, west, and south), population density, caffeine intake (<100 mg/day vs. over 100 mg/day, except in caffeine stratified analyses) and ibuprofen use (ever/never user, except in ibuprofen stratified analyses).

^per 10 μg/m^3^.

Our analyses did not differ notably with additional adjustment for tract-level income and housing value. Analyses using pollution values in 2000 as the exposure and lagging PM exposure by 5 years with respect to PD onset also did not differ materially from the primary analyses. Analyses that also included cases that did not confirm their diagnosis of PD cases were similar to the main analyses. We conducted additional sensitivity analyses among non-movers (Table 4), the results of which were not significantly different from those of our main analyses.

**Table 4:** **Exposure to PM**_**10** ,_ **PM**_**2.5** ,_ **and PM**_**10**–**2.5**_ **and risk of PD in the Nurses**’ **Health Study among non**-**movers**, **1990**–**2008** (**N** = **80**,**544 women at baseline who did not move during the study**)

	Multivariate ^a^				
	PY	Cases	RR	95% CI	P-trend
**Quartiles of PM**_**10**_					
3.8-21.0 μg/m^3^	395353	123	1.00	Ref	
21.0– 24.3 μg/m^3^	395383	115	1.09	(0.84, 1.41)	
24.3 – 28.3 μg/m^3^	395419	100	1.00	(0.76, 1.33)	
28.3 – 88.3 μg/m^3^	395426	90	0.98	(0.76, 1.33)	0.92
**Continuous^** **PM**_**10**_	1581580	430	0.99	(0.89, 1.09)	
**Quartiles of PM**_**2.5**_					
1.2-12.6 μg/m^3^	395343	110	1.00	Ref	
12.6-15.0 μg/m^3^	395383	111	1.10	(0.83, 1.45)	
15.0-17.4 μg/m^3^	395409	121	1.25	(0.94, 1.65)	
17.4-73.9 μg/m^3^	395446	88	1.11	(0.81, 1.52)	0.43
**Continuous^** **PM**_**2.5**_	158446	430	1.08	(0.81-1.45)	
**Quartiles of PM**_**10**–**2.5**_					
0-7.2 μg/m^3^	395375	132	1.00	(Ref)	
7.2-9.2 μg/m^3^	395394	106	0.93	(0.71, 1.21)	
9.2-12.0 μg/m^3^	395397	96	0.90	(0.68, 1.20)	
12.0-67.1 μg/m^3^	395395	96	0.90	(0.63, 1.28)	0.64
**Continuous^** **PM**_**10**–**25**_	1937996	430	0.95	(0.85-1.05)	

*PY*: person years; *RR*: relative risk; 95% *CI*: 95 percent confidence interval.

^a^adjusted for age, smoking, region (northeast, midwest, west, and south), population density, caffeine intake (<100 mg/day vs. over 100 mg/day) and ibuprofen use (ever/never user).

^per 10 μg/m^3^.

## Discussion

In this study, we did not observe a statistically significant increase in PD risk associated with exposure to ambient air pollution measured as cumulative exposure to PM_2.5_, PM_10_ and PM_10–2.5_ at the residential address. We did not observe any interaction with smoking or caffeine for any of the size fractions. An interaction was observed between PM_10_ and PM_10–2.5_ and use of ibuprofen, but this this was an unexpected finding and could have occurred by chance.

PM_10_ includes all particles smaller than 10 microns in diameter; a size considered to be small enough to pass through the throat or the nose and enter the lungs, thus potentially causing harm to human health. PM_10_ includes inhalable coarse particles (PM_10–2.5_) and fine particles (PM_2.5_). Inhalable coarse particles, PM_10–2.5_ comes primarily from agricultural, mining and construction sources [23]. Fine particles, PM_2.5,_ consists primarily of combustion particles from the burning of coal, wood and fuel oil and from motor vehicle emissions [23]. Exposure to PM has been associated with many harmful effects on human health. The Harvard Six Cities Study [1] and the American Cancer Society Study [2] were the first major studies to link exposure to air pollution to all cause mortality (The RR for all cause mortality in the extended follow-up of the Harvard Six Cities Study was 1.16 (95% CI: 1.07-1.26) [24]). Since then, many more studies have documented the detrimental effects of chronic exposure to air pollution on human health and survival [24, 25].

Prior work on air pollution and PD has been limited. Finkelstein and colleagues [26], found that although markers of traffic derived air pollution did not predict PD risk, risk was increased among participants with higher Manganese (Mn) exposure (RR: 1.03; 95% CI: 1.00-1.07) for each 10 ng/m^3^ increase in Mn concentration) [26]. In a recent study that used GIS to link airborne metal exposure data throughout the US to the Medicare beneficiaries database, Willis and colleagues found a higher incidence of PD in urban counties with higher industrial release of copper and manganese [14]. Also, a number of studies have linked occupational manganese exposure to risk of Parkinsonian syndromes [27, 28] although this is thought to be distinct from PD [29].

Our study’s estimates of PM air pollution have an advantage over what was done in prior studies because the measures are based on a comprehensive prediction model of PM. This model allows us to estimate air pollution levels for an entire cohort of non-occupationally exposed participants at each participant’s residential address. Also, our air pollution models used GIS-based spatio-temporal statistics with GIS covariates that allow us to account for small-scale variations in pollution around each woman’s address and to estimate the pollution level for each address that each study participant has reported during the study period, thus giving us a more accurate measure of exposure to air pollution over time than past studies.

Animal studies suggest that PM may access the brain either through the nasal pathway or through the circulation [15, 16, 23, 30]. Fine particles in the central nervous system have been associated with increased brain inflammation in several studies [31–33]. In one study, levels of several pro-inflammatory cytokines were elevated in the brains of mice exposed to high levels of particulate matter compared to controls [31]. In another study in rats, exposure to diesel exhaust was associated with an increase of a number of inflammatory factors, including whole-brain IL-6 and TNF α, among others [34]. Furthermore, a study of Mexico city residents, showed higher levels of neuroinflammation of the olfactory bulb (as indicated by higher levels of COX 2 and IL1β) and higher concentrations of metals associated with PM (including manganese, nickel and chromium) among inhabitants of urban areas [35]. A study using autopsies of brains from children and young adults in Mexico City, Mexico found elevation of indices on neuroinflammation and oxidative stress in the brains of participants exposed to high levels of particulate matter [36]. Higher levels of inflammatory factors in the brain have been linked with increased risk of PD in several studies. PD patients had significantly higher levels of oxidized –LDL and high-sensitivity of C-reactive protein (both markers of inflammation) compared to controls in one study [5, 7], and elevated levels of Il-6, but not CRP in another study [7].

Measurement of long-term exposure to air pollution is prone to substantial error, which under most circumstances would tend to attenuate an association with PD risk. In this study, we only had information on air pollution exposure from 1988 onwards, due to the availability of the modeled air pollution data. This period covers only adulthood exposure for our study population. It is conceivable, although this has not been determined, that the relevant etiological period may include exposure during childhood or, more generally, for a longer period of time than we were able to capture in this study. To address this issue, we conducted a sensitivity analysis restricted to women who did not move during the study (with the presumption that those women were also more likely to maintain a single residence prior to study baseline) (Table 4). In analyses restricted to women who maintained the same State and County (430 PD cases) and in analyzes among women who maintained the same latitude and longitude (291 PD cases) throughout the study, our results still did not indicate a significant association between exposure to PM air pollution and risk of PD (results not shown). Furthermore, because direct measurement data on PM_2.5_ was only available post-1999, we conducted sensitivity analyses starting follow-up in 1999 and only using the 311 PD cases onset after January 1 1999. The results of these analyses did not differ significantly from our main results.

Another limitation of our study is in the exposure assessment: we did not have personal air pollution measurements in this study or indoor air pollution measures and we do not know how much time people spend indoors vs. outdoors at their address. Due to the large scale of this study, it was only possible to use indirect measures of air pollution, collected via GIS-based modeling of pollution based on values measured by monitors throughout the US. Although the models used to estimate PM in the NHS have been shown to have little bias and high precision, [37, 38] especially when compared with other methods of estimating air pollution [18, 19], some misclassification of the biologically relevant levels of individual exposure is inevitable and could have attenuated the association between air pollution and PD risk. Finally, as in most epidemiological studies of a rare disease such as PD, power and sample size restrict our ability to detect associations - in this study we had 80% power to detect relative risks of 1.45 or above, comparing the highest quartile of pollution to the lowest quartile, assuming a 2-sided test with an alpha of 0.05. We expect that associations below this threshold would have been seen as suggestive, although not statistically significant findings. Also, there is also potential for occupational exposure to air pollution with the nursing occupation, which we were unable to account for in this study. Finally, we only had information on residential address, and were not able to account for exposure to ambient air pollution during the time that our study participants spent at work.

The strengths of this study include its’ large size, a long and prospective follow-up, a high response rate and the availability of PM_10_, PM_2.5_, PM_10–2.5_ estimates at each woman’s residential address on a monthly basis throughout the study. The pollution data was available throughout the continental US, allowing for sufficient variability to detect a potential contrast in the analytical categories as well as representativeness of the pollution levels to which residents of the US are commonly exposed.

## Conclusion

In summary, overall the results of this large cohort study of female nurses do not support an effect of air pollution on PD risk.

## Abbreviations

AQS: Air quality system
CI: Confidence intervals
EPA: Environmental Protection Agency
GIS: Geographic information system
IMPROVE: Interagency Monitoring of Protected Visual Environments
IRB: Institutional Review Board
Mn: Manganese
NHS: Nurses’ Health Study
PD: Parkinson’s disease
PM: Particulate matter
RR: Relative risk
US: United States.
